# P-14. Vaccine Effectiveness and Safety of Recombinant Zoster Vaccine in Inflammatory Bowel Disease Patient Populations Aged 50 Years and Older

**DOI:** 10.1093/ofid/ofae631.224

**Published:** 2025-01-29

**Authors:** Emily Rayens, Lina S Sy, Lei Qian, Jun Wu, Bradley Ackerson, Yi Luo, Yanjun Cheng, Avanish Patel, Zendi Solano, Justine De Jesus, Britta Amundsen, Ana Florea, Jennifer H Ku, Elizabeth Chmielewski-Yee, Driss Oraichi, Harry Seifert, Huifeng Yun, Hung Fu Tseng

**Affiliations:** Kaiser Permanente Southern California, Pasadena, California; Kaiser Permanente Southern California, Pasadena, California; Kaiser Permanente Southern California, Pasadena, California; Kaiser Permanente Southern California, Pasadena, California; Kaiser Permanente Southern California, Pasadena, California; Kaiser Permanente Southern California, Pasadena, California; Kaiser Permanente South California, San Gabriel, California; Kaiser Permanente Southern California, Pasadena, California; Kaiser Permanente Southern California, Pasadena, California; Kaiser Permanente Southern California, Pasadena, California; Kaiser Permanente Southern California, Pasadena, California; Kaiser Permanente Southern California, Pasadena, California; Kaiser Permanente Southern California, Pasadena, California; GSK, Rockville, Maryland; GSK, Rockville, Maryland; GSK, Rockville, Maryland; GSK, Rockville, Maryland; Kaiser Permanente Southern California, Pasadena, California

## Abstract

**Background:**

Adults with inflammatory bowel disease (IBD) are at higher risk of herpes zoster (HZ) compared to the general population. In 2021, the Advisory Committee on Immunization Practices recommended 2 doses of recombinant zoster vaccine (RZV) for the prevention of HZ and its complications in immunocompromised adults ≥19 years. In this study, we evaluated the vaccine effectiveness (VE) of RZV and risk of flares in adults ≥50 years with IBD.

**Methods:**

To evaluate VE, we conducted a retrospective matched cohort analysis at Kaiser Permanente Southern California. We included individuals ≥50 years with IBD who received 2 doses of RZV at least 28 days apart during 04/01/2018–12/31/2021 and matched up to 1:3 to RZV unvaccinated individuals on condition (ulcerative colitis, Crohn’s disease), age, sex, race/ethnicity, IBD medication type, and index date (date of second dose among vaccinated individuals; same date assigned to unvaccinated match). Follow-up started the 31^st^ day after the index date and ended at the earliest date of HZ occurrence, death, disenrollment, HZ vaccination or 12/31/2022. Stratified Cox proportional hazards regression was used to estimate adjusted hazard ratios and VE against HZ, identified by ICD-10 codes and antiviral medications. Using self-controlled case series analysis, we calculated the incidence of IBD new-onset flares in the risk (30 days after each dose) and comparison periods (90 - 61 days prior and 31 - 60 days after each dose) among patients with ≥1 RZV dose. The relative risk (RR) of IBD flare was estimated using conditional Poisson regression. All flares were identified through chart review.

**Results:**

Of adults ≥50 years with IBD (n=872 2-dose vaccinated, 2550 unvaccinated), 55.4% were female, 74.0% were non-Hispanic white, and mean age was 68.4 years (standard deviation [SD] 9.0). Overall adjusted VE in preventing HZ was 65.1% (95% CI 24.8–83.8%). Among 1199 adults ≥50 years old with IBD receiving ≥1 dose of RZV (53.8% female, 69.6% non-Hispanic white, mean age 68.2 years [SD 9.1]), there was no increased risk of IBD flare within 30 days after RZV vaccination (RR: 0.80; 95% CI 0.47–1.35).

**Conclusion:**

In adults aged ≥50 with IBD, 2 doses of RZV is effective in preventing HZ with no new safety concerns; RZV vaccination could benefit IBD patients who are at increased risk of HZ.

**Disclosures:**

**Emily Rayens, PhD, MPH**, GSK: Grant/Research Support|Moderna: Grant/Research Support **Lina S. Sy, MPH**, Dynavax: Grant/Research Support|GlaxoSmithKline: Grant/Research Support|Moderna: Grant/Research Support **Lei Qian, PhD**, Dynavax: Grant/Research Support|GlaxoSmithKline: Grant/Research Support|Moderna: Grant/Research Support **Jun Wu, MD, MS**, Genentech: Grant/Research Support|GlaxoSmithKline: Grant/Research Support|VoxelCloud: Grant/Research Support **Bradley Ackerson, MD**, Dynavax: Grant/Research Support|GlaxoSmithKline: Grant/Research Support|Moderna: Grant/Research Support|Pfizer: Grant/Research Support **Yi Luo, PhD**, GlaxoSmithKline: Grant/Research Support|Moderna: Grant/Research Support|Pfizer: Grant/Research Support **Yanjun Cheng, Master of Science**, GlaxoSmithKline: Grant/Research Support **Avanish Patel, MD**, GlaxoSmithKline: Grant/Research Support **Zendi Solano, BS**, Dynavax: Grant/Research Support|Gilead: Grant/Research Support|GlaxoSmithKline: Grant/Research Support **Justine De Jesus, MPH**, GlaxoSmithKline: Grant/Research Support **Britta Amundsen, MA**, Gilead: Grant/Research Support|GlaxoSmithKline: Grant/Research Support **Ana Florea, PhD, MPH**, Gilead: Grant/Research Support|GSK: Grant/Research Support|Moderna: Grant/Research Support|Pfizer: Grant/Research Support **Jennifer H. Ku, PhD MPH**, GSK: Grant/Research Support|Moderna: Grant/Research Support **Elizabeth Chmielewski-Yee, PhD, MPH**, GSK: Employee of GSK|GSK: Stocks/Bonds (Public Company) **Driss Oraichi, PhD**, GSK: I am a full time employee of GSK|GSK: Stocks/Bonds (Private Company) **Harry Seifert, MD. MSCE, FISPE**, GSK: full-time employee|GSK: Stocks/Bonds (Public Company) **Huifeng Yun, MD, PhD**, GSK: fulltime employee of GSK|GSK: Stocks/Bonds (Public Company) **Hung Fu Tseng, PhD MPH**, GlaxoSmithKline: Grant/Research Support|Moderna: Grant/Research Support

